# Announcing the 2016 *Toxins* Travel Awards for Post-Doctoral Fellows and Ph.D. Students

**DOI:** 10.3390/toxins8020041

**Published:** 2016-02-06

**Authors:** Vernon L. Tesh

**Affiliations:** Department of Microbial and Molecular Pathogenesis, Medical Research and Education Building, Room 3002, College of Medicine, Texas A&M University System Health Science Center, 8447 State Highway 47, Bryan, TX 77807, USA; tesh@medicine.tamhsc.edu

With the goal of promoting the development of early career investigators in the field of toxinology, *Toxins* welcomed applications for the 2016 *Toxins* Travel Awards for post-doctoral fellows and Ph.D. students. We received nearly 100 completed applications for the awards, and the overall quality of all the applications was outstanding. While this bodes well for the future of toxinology, it certainly made for a challenging task for the editors selecting the two finalists. On behalf of the editors of *Toxins*, I am pleased to announce the winners of the inaugural *Toxins* Travel Awards for 2016.

The *Toxins* Travel Awards are granted to Dr. Kartik Sunagar, Marie Skłodowska-Curie Fellow in Dr. Yehu Moran’s laboratory at The Hebrew University of Jerusalem, Israel, and to Dr. Philipp Wiemann, a post-doctoral researcher in Dr. Nancy Keller’s laboratory at the University of Wisconsin-Madison, USA. They will each receive 800 CHF to help support travel to present at scientific conferences in 2016.


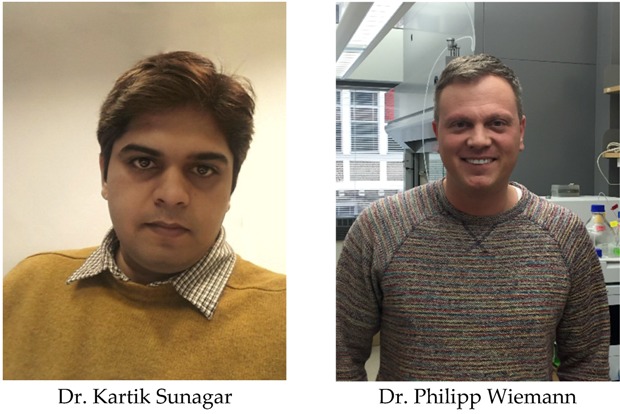


Dr. Kartik Sunagar received his Ph.D. in Evolutionary Biology from the University of Porto in Portugal in 2013. In 2014, Dr. Sunagar was awarded the Marie Skłodowska-Curie Individual Fellowship, one of the European Union’s most prestigious fellowships for post-doctoral researchers. He joined Dr. Moran‘s laboratory, where he studies animal venoms to understand various aspects in evolutionary biology and ecology, with particular emphasis on molecular evolution, predator–prey interactions, mechanisms of toxic action and the role of environmental and ecological factors in driving the evolution of venom—nature’s most complex biochemical cocktail. In Dr. Moran’s letter of support, he wrote: "During the time Kartik spent in my lab he demonstrated originality, intelligence and research skills that I rarely encountered in a young researcher of a similar career stage." Dr. Sunagar has published 15 peer-reviewed research articles and five reviews or book chapters, including six papers in *Toxins*. He has been invited to present at a number of international conferences, including a recent oral presentation at the 18th World Congress of the International Society on Toxinology. Dr. Sunagar will use the *Toxins* Travel Award to attend the annual conference of the Society for Molecular Biology and Evolution on the Gold Coast in Queensland, Australia, July 3–7, 2016. 

Dr. Philipp Wiemann received his Ph.D. in Food Chemistry *summa cum laude* from Westfälische Wilhelms-Universität Münster, Germany in 2010 where he worked in the laboratories of Prof. Hans-Ulrich Humpf and Prof. Bettina Tudzynski. After a brief post-doctoral fellowship in Dr. Tudzynski’s laboratory, Dr. Wiemann joined the laboratory of Dr. Nancy P. Keller at the University of Wisconsin-Madison. In the Keller laboratory, he has been working with Aspergillus species, including the opportunistic human pathogen *A. fumigatus*, and the aflatoxin producing plant pathogen *A. flavus*. His research mainly focuses on a specific natural product of *A. fumigatus*, called hexadehydroastechrome, that, when over-produced, contributes to virulence in mice. In Dr. Keller’s letter of support, she wrote: "Philipp has been a fantastic post-doctoral scientist in my lab, excelling in every aspect of professional life from research to ethical standards and lab citizenship." Dr. Wiemann has co-authored 18 peer-reviewed research publications including a recent paper in *Toxins*. He is a frequent invited presenter at international meetings on fungal genetics. Dr. Wiemann will use the *Toxins* Travel Award to attend the Gordon Research Conference on Cellular and Molecular Fungal Biology in Holderness, New Hampshire, USA, June 19–24, 2016.

The editors, managing editors, and editorial board members congratulate Drs. Sunagar and Wiemann on winning the 2016 *Toxins* Travel Awards and we are grateful to MDPI for their generous support of the awards this year and in the future. 

